# Optimization of DNA extraction methods from pig farm wastewater for pathogen detection using metagenomic sequencing

**DOI:** 10.1099/mgen.0.001532

**Published:** 2025-10-24

**Authors:** Manavi Muralidhar, Muhammad Noman Naseem, Jose Ramon Botella Mesa, Lida Omaleki, Conny Turni

**Affiliations:** 1 Queensland Alliance for Agriculture and Food Innovation, The University of Queensland, St Lucia, QLD, 4067, Australia; 2 School of Agriculture and Food Sustainability, The University of Queensland, St Lucia, QLD, 4067, Australia

**Keywords:** DNA extraction, Oxford Nanopore Technology (ONT) sequencing, pathogen surveillance, piggery wastewater

## Abstract

Wastewater can be a useful sample to monitor disease outbreaks in the community, as it was demonstrated during the recent Severe acute respiratory syndrome coronavirus 2 pandemic. Due to housing conditions, diseases can rapidly spread within pig herds, resulting in high mortalities and significant economic losses. Monitoring piggery wastewater using Oxford Nanopore Technology’s (ONT) sequencing platform combined with metagenomic analysis can provide early disease detection to deploy preventative measures. Nevertheless, obtaining DNA of the required purity and integrity from piggery wastewater is a major challenge. This study aims to identify and optimize the most effective method for obtaining high-quality and quantity DNA, which can be used in downstream applications for pathogen detection. Six DNA extraction protocols were tested on piggery wastewater samples and evaluated based on yield and overall DNA quality. The three best-performing methods, using commercially available kits (QIAGEN QIAamp® PowerFecal® Pro, QIAGEN DNeasy® PowerLyzer® PowerSoil® and Macherey-Nagel NucleoSpin® Soil), were then used to extract DNA from piggery wastewater samples spiked with a mock community composed of known pig pathogens. The extracted DNA samples were then sequenced on the ONT platform, and the effectiveness of the methods was evaluated using kraken2 taxonomic classifier and an in-house database. Results demonstrated that the optimized QIAGEN PowerFecal® Pro protocol was the most suitable and reliable extraction method. Overall, this study highlights the importance of determining the optimal DNA extraction method in effective disease surveillance using a complex environmental sample and takes an important step in making metagenomic disease surveillance a practical reality.

**Impact Statement**

Effective surveillance and early disease detection in livestock – critical not only for global food security but also for addressing the One Health challenges – are fundamental to mitigating outbreaks. Through this work, we evaluated six DNA extraction methods for their performance with piggery wastewater, revealing significant discrepancies in their ability to recover high-quality bacterial DNA from this complex matrix. Based on these findings, we identified three extraction kits that performed better and tested them for a further deeper analysis. By spiking piggery wastewater with a known mock community, we were able to demonstrate biases in DNA extraction processes, highlighting the variability that can influence pathogen detection. This work enabled us to select and optimize the most effective DNA extraction method for pathogen surveillance in piggery wastewater. From a broader perspective, our findings emphasize that to establish metagenomic surveillance as a routine tool, optimization and standardization of extraction methods for each matrix is critical to ensure reliable results that can drive early disease detection and intervention.

## Data Summary

All sequences have been deposited to the Sequence Read Archive (SRA) under BioProject number PRJNA1179682 with accession numbers SRX26565502, SRX26565503, SRX26565504, SRX26565505, SRX26565506, SRX26565507, SRX26565508, SRX26565509, SRX26565510, SRX26565511, SRX26565512 and SRX26565513.

## Introduction

Pathogen surveillance enables timely disease detection, potentially preventing major outbreaks [1]. Even during active outbreaks, surveillance practices can provide essential information to evaluate the effectiveness of the deployed control measures. Wastewater has been used for many years in the detection of polio viruses [2,4]. More recently, wastewater was used during the Severe acute respiratory syndrome coronavirus 2(SARS-CoV-2) viral pandemic for pathogen surveillance to monitor population health and disease spread [5,6]. Using next-generation sequencing in wastewater, Crits-Christoph *et al*. (2021) first detected the presence of SARS-CoV-2 variants [7], and the method has now been expanded to detect other pathogens in wastewater [8]. In principle, the same surveillance strategy can be used to monitor diseases in animals, such as pigs, that are usually housed in close proximity. Wastewater from piggeries has been previously used to study the movement of mobile genetic elements through the treatment process [9] and to investigate the organisms involved in wastewater treatment [10].

Wastewater is a complex environmental matrix that can contain microorganisms, nutrients, organic matter, metals and many other substances [11]. The negative effects of these inhibitors for downstream digital PCR and Illumina next-generation sequencing have been described previously [12]. Therefore, it is imperative to efficiently remove these inhibitors while retaining DNA integrity. Previous work utilizing nanopore sequencing to analyse human wastewater or sewage has used a combination of a bead beating step together with specialized environmental soil or water kits [13,15], while a study on metagenomics of pig wastewater used a filter step and a soil kit to purify DNA from wastewater [16]. In addition to purity of a sample, it is also important to consider DNA extraction biases that can occur in microbiome studies [17]. A previous study comparing DNA extraction methods for use with nanopore sequencing illustrates with the help of a mock community that different extraction methods do exhibit biases towards certain species [18]. This emphasizes the importance of using known bacteria in a matrix to correctly evaluate biases in an extraction method.

In this study, we compared six extraction methods for their ability to extract DNA from piggery wastewater based on yield and DNA quality. The best-performing methods were then used to analyse wastewater samples containing a spike-in mock community of respiratory bacterial pathogens commonly found in pigs by nanopore sequencing.

## Methods

This study is divided into two parts (Table 1). In the first part, different extraction methods were compared based on the quantity and quality of the extracted DNA from piggery wastewater. Extraction methods that resulted in a higher quantity and quality of yielded DNA in the first part were then selected for the second part of the study. In the second part, the selected methods were used to extract DNA from piggery wastewater samples that were spiked with a known mock community of organisms. The extracted DNA from the second part was then sequenced on an Oxford Nanopore Technologies (ONT) (Oxford, UK) MinION platform, and the recovery of the spiked organisms was compared across methods.

**Table 1.** Summary of the sample preparation methods for DNA extraction

Part	Sequencing	Farm	Volume taken (ml)	Replicates per extraction method
One	No	A	40	3
		C	10	3
		D	40	3
Two	Yes	E	10	4

### Sample collection

Wastewater samples were collected from four piggeries (henceforth referred to as farms A, C, D and E) in Queensland, Australia. In farms C, D and E, samples were collected at the wastewater collection ponds, while in farm A, they were collected under the pig sheds. Sheds are areas housing the pigs, and the waste from the floors of these sheds is collected in drains under the floor. Wastewater collection ponds are outdoor ponds where wastewater from all sheds is collected. Collection was done using sterile containers, with the samples transported on ice to the laboratory where they were stored at −20 °C until further analysis. Wastewater collected from farms A, C and D was used for the first part of the study while wastewater from farm E was used for the second part of the study (Table 1).

### Sample preparation

To prepare the samples for DNA extraction, 10 ml of collected wastewater samples was used from farms C and E, while 40 ml was used from farms A and D. The discrepancy in volume was due to higher particulate matter content in the wastewater from farms C and E compared to that from farms A and D. A summary of the volumes used from each farm is provided in Table 1. Samples were centrifuged at 46 ***g*** for 1 min to settle the heavier solids. The supernatant was then centrifuged at 4,550 ***g*** for 30 min. After the second round of centrifugation, the supernatant was discarded, and the pellet was weighted and stored at −20 °C until further analysis. Pellets from farm A ranged in weight from 0.37 to 0.67 g, farm C from 0.39 to 0.59 g and farm D from 0.46 to 0.55 g. Before DNA extraction, the pelleted material was thawed to room temperature and reconstituted in 500 µl of Merck (Darmstadt, Germany) Milli-Q® water. A total of 0.3 g of the homogenized material (henceforth referred to as homogenate) was used for extraction.

### DNA extraction

The extractions in this section were conducted using the homogenate from farms A, C and D.

#### QIAamp® PowerFecal® Pro DNA kit

This method is henceforth referred to as PF. The kit was purchased from QIAGEN (Hilden, Germany), and the protocol was followed as per the manufacturer’s instructions, with a few modifications. Instead of the kit recommended 800 µl, 500 µl of CD1 lysis buffer was added. Samples were mechanically lysed using the Vortex-Genie 2 (Scientific Industries Inc., NY, USA) at maximum speed for 10 min. The wash step with C5 solution was modified into two steps of 250 µl each, followed by incubation on ice for 5 min and then centrifugation at 13,000 ***g***. Following the final wash step, lids of the spin column were left open for 10 min to ensure the evaporation of all residual ethanol. At the 5 min mark, Solution C6 was added to the column. DNA was eluted in a 50 µl volume.

#### PureGene® Tissue Core Kit

This method is henceforth referred to as PG. The kit was purchased from QIAGEN and the protocol was followed as per the manufacturer’s instructions, with a few modifications. Following the cell lysis step, 20 µl of proteinase K was added to the samples. The samples were then incubated at 56 °C at 300 r.p.m. for 2 h using an Eppendorf® (Hamburg, Germany) ThermoMixer® Comfort. Following DNA precipitation, samples were left at room temperature overnight in 50 µl of DNA hydration buffer as per the manufacturer’s suggestions.

#### In-house method

An in-house method was developed for DNA extraction from the piggery effluent and henceforth referred to as IH. Homogenate was made from pelleted material as described above and dissolved in 350 µl of lysis buffer (0.1M EDTA, 0.1M Tris and 0.5 M NaCl). The mix was then transferred into a fresh 1.5 ml Eppendorf tube, and 50 µl of 10% SDS was added and vortexed for 5 s. All incubation steps were done on an Eppendorf® ThermoMixer® Comfort. Samples were then incubated at 98 °C for 20 min and vortexed every 5 min for 3 s. They were then placed on ice for 5 min, followed by a second incubation at 98 °C for 5 min. After cooling on ice for another 5 min, samples were spun down at 18,620 ***g*** for 5 min using a DLab (Beijing, China) D3024 centrifuge. The resulting supernatant was then transferred into a 2 ml LoBind tube (Eppendorf®). For DNA purification, AMPure XP beads (Beckman Coulter, Brea, CA, USA) were added in a 1:1 ratio, with a maximum of 400 µl per sample. Samples were incubated at 20 °C for 15 min with 300 r.p.m. shaking. The tubes were then placed on an Invitrogen™ (Waltham, MA, USA) DynaMag™−2 magnetic rack to separate the liquid, which was subsequently removed. Beads were washed twice with 400 µl of freshly prepared 70% ethanol. After the second wash, the lids of the tubes were left open for 10 min to allow for the evaporation of any residual ethanol. For eluting the DNA, the tubes were removed from the magnetic rack and 50 µl of Milli-Q water was added, followed by incubation at 37 °C for 15 min. Following this, the tubes were once more placed on a magnetic rack, and the resultant eluted DNA was transferred to a new tube.

#### NucleoSpin® Soil

This method is henceforth referred to as NS. The NucleoSpin® Soil kit from Macherey-Nagel (Dueren, Germany) was used according to the manufacturer’s instructions, with a few modifications. Following the wash step with the SW2 buffer, the lids of the spin column were left open for 5 min to allow the evaporation of any residual ethanol. DNA was eluted in a 50 µl volume of the provided elution buffer.

#### PrepMan™ Ultra

This method is henceforth referred to as PM. The PrepMan™ Ultra reagent from Applied Biosystems™ (Waltham, MA, USA) was used, and the protocol was followed according to the manufacturer’s instructions with a final elution volume of 50 µl.

#### DNeasy® PowerLyzer® PowerSoil®

The DNeasy® PowerLyzer® PowerSoil® kit (QIAGEN) was used as per the manufacturer’s instruction for this method which henceforth is referred to as PS. The few modifications to the manufacturer’s instructions are described below. Samples were mechanically lysed using the Vortex-Genie 2 (Scientific Industries, Inc.) at maximum speed for 10 min. Following the final wash step, lids of the spin column were left open for 5 min before adding the C6 elution buffer to ensure the evaporation of all residual ethanol. Once the buffer was added to the column, the lids were once again left open for 5 min. DNA was eluted in a 50 µl volume of elution buffer.

### Calculation of DNA quantity and quality

DNA concentration was measured using 1 µl of eluted DNA on an Invitrogen™ QUBIT™ 4 using the dsDNA Broad Range kit following manufacturer’s instructions. Standards were prepared freshly and ran with every set of measurements. DNA quality was checked using 2 µl of eluted DNA on a NanoDrop 8000 instrument (Thermo Fisher Scientific, MA, USA).

### Preparation of a mock community

A mock community of the relevant respiratory bacterial pathogens that most commonly affect piggeries at the grower stage was made. The Shope 4074 strain (CCUG 12837) of *Actinobacillus pleuropneumoniae*, S 735 strain (CCUG 7984) of *Streptococcus suis* and strain P-2237 (CCUG 25985) of *Pasteurella multocida* were used in the present study. *S. suis* and *P. multocida* were revived from the culture collection available in the laboratory on sheep blood agar. *A. pleuropneumoniae* was revived on blood agar/selective nutrient (BA/SN) plates.

A 6×10^8^ c.f.u. ml^−1^ suspension was made from each of the bacteria of interest by adding colonies of overnight culture to PBS and comparing turbidity against a McFarland Standard 2.0. A 1:10 serial dilution of each bacterium was made down to a 6×10^1^ c.f.u. ml^−1^ suspension. To obtain an estimate of the concentration used for the community, 100 µl of the suspensions at the 6×10^4^ to 6×10^2^ c.f.u. ml^−1^ dilutions was cultured on the appropriate plates noted in this section. After a 24-h incubation, colonies were counted to determine an approximate number of c.f.u. spiked-in.

The mock community was prepared by mixing 1 ml of each of the 6×10^8^ c.f.u. ml^−1^ bacterial suspension (*A. pleuropneumoniae*, *S. suis* and *P. multocida*) in a tube.

### Sample preparation for sequencing

This part of the experiment was done using the homogenate from farm E only. A volume of 10 ml of wastewater from farm E was pelleted by centrifugation as previously described. Pellets’ weight ranged from 0.24 to 0.26 g. A total of 12 wastewater pellets were prepared with six used as control, while the other six were ‘spiked’ with the prepared mock community as described above. Pellets were homogenized after adding 100 µl of water, creating what is henceforth referred to as the ‘homogenate’. The six control samples contained 300 µl of the homogenate, while the six spiked samples consisted of 290 µl of the homogenate and 10 µl of the mock community.

Following this, DNA was extracted from two control and two spiked samples for each method – PF, NS and PS – as outlined in the previous section. The quality and quantity of the extracted DNA were checked, and those that did not meet the absorbance requirements were passed through the Zymo Research (Irvine, CA, USA) OneStep PCR Inhibitor Removal kit.

### Library preparation for sequencing

DNA library was prepared using the SQK-NBD114.24 gDNA (genomic DNA) ligation sequencing kit by ONT, as per the manufacturer’s instruction with few modifications as per a publication by Nguyen *et al*. [19]. The incubation times for all the library preparation steps (end-repair, barcoding and adapter ligation and clean-up) were extended to 30 min. Incubation time for elution of DNA after end-repair was extended to 10 min, and incubation for elution at the end of barcoding and adapter ligation and clean-up steps was extended to 15 min.

DNA concentrations were measured following each step of the library preparation using the QUBIT™ dsDNA Broad Range kit. Measurements obtained after the adapter ligation step were used to determine the molarity of the prepared library, assuming a length of 2,000 bp. This assumption was made based on previous sequencing attempts using the PF kit and samples that were previously sent for fragment analysis (data not shown). Calculation for the number of moles being loaded was made using an online calculator (https://www.promega.com.au/resources/tools/biomath/). The final prepared library was loaded onto an ONT R10.4.1 flow cell. Sequencing was conducted using a MinION™ Mk1C (ONT) device with MinKNOW v23.04.5. Sequencing was conducted until ~1 giga base pair (Gb) of data was collected per barcode.

### Bioinformatic analysis

Data were basecalled and demultiplexed using the ONT basecalling software Dorado (https://github.com/nanoporetech/dorado; v0.5.2) retaining reads with a Q-score of 7 and above, and with the ‘sup-accurate’ model. Reads were then filtered using Filtlong (https://github.com/rrwick/Filtlong; v0.2.1-gcc-10.3.0) to remove reads under 100 bp. The filtered reads were then classified using kraken2 (v2.1.2) [20], with all default settings and against a database that was built using the kraken2 bacterial reference library downloaded in June 2024. Functional analysis was performed using DIAMOND (v2.1.7) and MEGAN7 [21] using the non-redundant protein database and following the setting for long read microbiome analysis mentioned in the publication by Bagci *et al*. Functional analysis was then performed using the SEED [22].

### qPCR for mock community

Species-specific quantitative polymerase chain reaction (qPCR) was performed for confirmation of the presence of the bacteria used to build the mock community. Protocols were followed from published papers for each of the bacterial species. Genes used were *apxIVA* for *A. pleuropneumoniae* [23], *sodA* for *P. multocida* [24] and *gdh* for *S. suis* [25]*.*

A standard curve was generated using DNA extracted from the pure cultures of each of the bacterial species used in the mock community. A 1 µl loopful of overnight culture was taken from the *A. pleuropneumoniae* and *P. multocida* plates and mixed in 100 µl of water. This mixture was then boiled at 100 °C for 10 min followed by incubation on ice for 5 min. This process was repeated a second time, following which the samples were spun at 18,000 ***g*** for 3 min. For extraction of *S. suis*, a loopful of culture was mixed with the lysis buffer from the DNeasy® Blood and Tissue kit. Extraction was carried out according to the manufacturer’s instructions. All DNA extracts were quantified using the Invitrogen™ QUBIT™ with the dsDNA Broad Range kit. A 1:10 dilution series was made using the extracted DNA from each organism and used as template in the relevant qPCR assays to generate the standard curve. All DNA extracts were diluted before being used as a template to allow for approximately 5–20 ng of template DNA. A no template control consisting of water was included with every run.

### Data analysis

Data were analysed using RStudio (v2023.12.0) running R v4.3.2. R packages phyloseq (v1.46.0) [26], vegan (v2.6.4) [27], biomformat (v1.30.0) [28], microbiomeutilities (v1.0.17) [29], Hmisc [30] and ggplot2 (v3.4.4) [31] were used for the data visualization and analysis. Galaxy Australia (v23.1.5.dev0) was used to run NanoPlot (v1.41.0+galaxy0) [32] for evaluating the quality of filtered reads. Microsoft Excel was used for the summary statistical analyses.

Graphs were generated on RStudio comparing the results of each method based on yield, *A*_260/280_ and *A*_260/230_ absorption ratios. A Kruskal–Wallis test was conducted to evaluate the overall differences between the extraction methods, and a Wilcoxon test was conducted to explore significant differences between the extraction methods. This was done by comparing each method against the extraction method that showed the highest DNA yield and the best *A*_260/280_ and *A*_260/230._

## Results

### DNA extraction from piggery wastewater using different methods

**Fig. 1.**
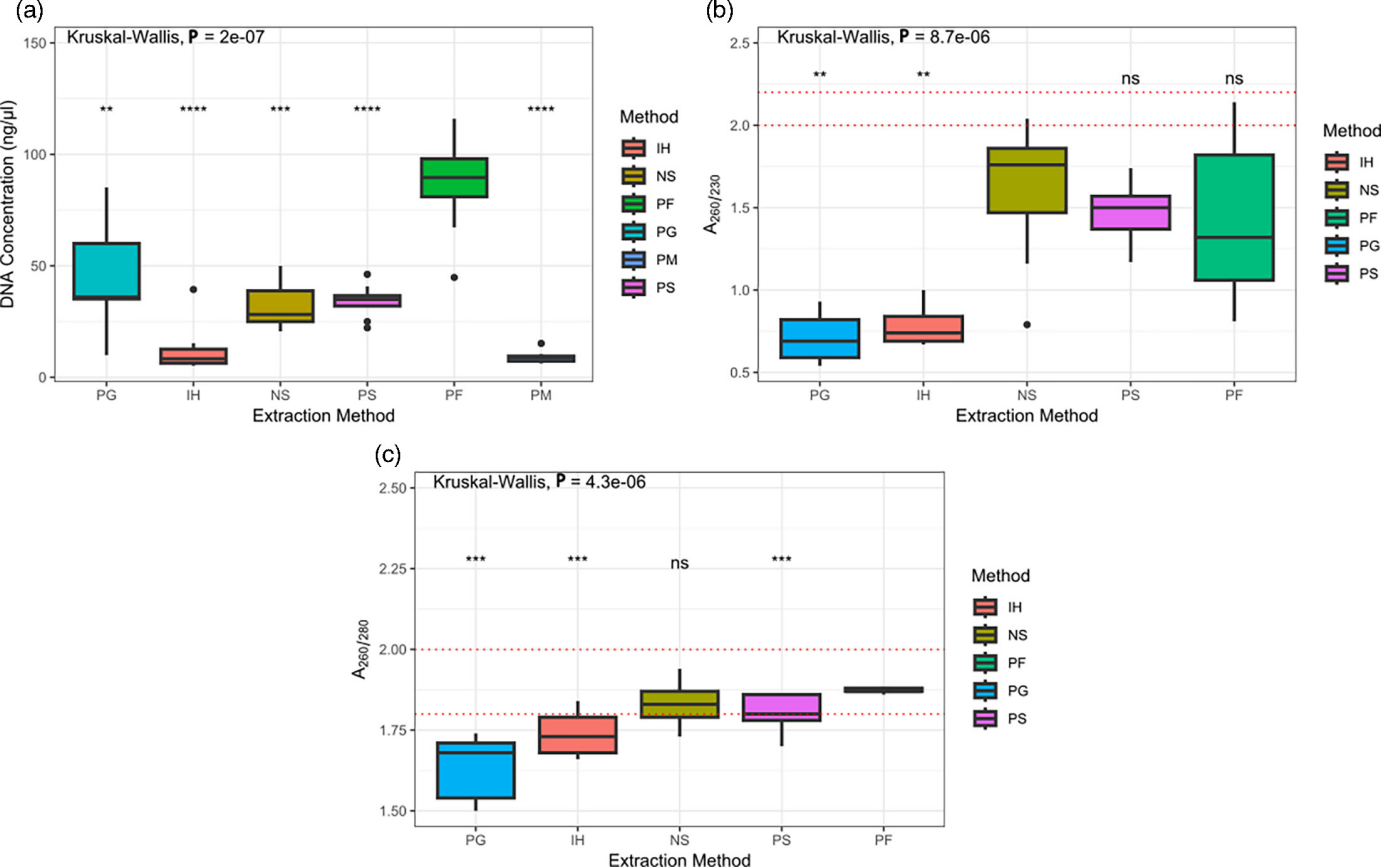
(a) DNA yields for all tested extraction methods. DNA quantity was measured using a QUBIT™ 4 fluorometer. A Wilcoxon test was performed by comparing the results of each extraction method with the PF method. (b) *A*_260/230_ absorbance ratios observed for all extraction methods. A Wilcoxon test was performed by comparing the results of each extraction method with the NS method. (c) *A*_260/280_ absorbance ratios observed for all extraction methods. A Wilcoxon test was performed by comparing the results of each extraction method with the PF method. Absorbance ratios were measured using a NanoDrop™ 8000 instrument. Overall statistical significance was calculated using a Kruskal–Wallis test. PF, QIAamp® PowerFecal® Pro DNA kit; PG, PureGene® Tissue Core Kit; PM, PrepMan™ Ultra; IH, in-house method; NS, NucleoSpin® Soil; PS, DNeasy® PowerLyzer® PowerSoil®. ns, not significant, *P*>0.05, *P<0.05, **P<0.01, ***P<0.001, ****P<0.0001.

Six different extraction protocols were used in this study consisting of five commercially available extraction kits and one in-house method. A summary of the yield and purity for each method has been provided in Fig. 1. The PM method produced very low DNA yields with a high level of particulate matter present in the final samples, making it impossible to determine the *A*_260/280_ and *A*_260/230_ absorbance ratios and was not used for further analysis.

All tested parameters indicated significant differences between the extraction methods as measured using a Kruskal–Wallis test (Fig. 1). With the exception of PG, *A*_260/280_ ratios for the remaining methods were close to 1.8, suggesting the presence of low protein contamination in the final DNA samples. In our experiments, none of the extracted DNA samples were able to reach the recommended absorbance ratio of 2–2.2 for the *A*_260/230_; however, the three bead-beading kits had the highest ratios. DNA from the PG and IH methods has extremely low *A*_260/230_ ratios of 0.71 and 0.78, respectively, indicating the presence of high levels of contaminants. Based on the above results, the NS, PS and PF methods were selected for further experiments.

### DNA extraction and library preparation for sequencing

A volume of 290 µl of the homogenized wastewater pellet from farm E (the pelleted material mixed with 100 µl of water) was spiked with 10 µl of a ‘mock community’ containing *P. multocida*, *A. pleuropneumoniae* and *S. suis*. The microbes were spiked by comparing bacteria suspended in PBS to a 2.0 McFarland standard. To confirm the quantities of each of the spiked-in bacterium, a 1:10 serial dilution of the PBS-suspended bacteria was made, and 100 µl of the 10^−4^, 10^−5^ and 10^−6^ dilutions was plated on the appropriate media (sheep blood agar for *P. multocida* and *S. suis* and BA/SN for *A. pleuropneumoniae*). The results of the plating indicated that ~2.7×10^5^ c.f.u. of *P. multocida*, 1.5×10^6^ c.f.u. of *A. pleuropneumoniae* and 2.4×10^6^ c.f.u. of *S. suis* were spiked in the wastewater. A total of 12 samples were sequenced – 6 controls (non-spiked) and 6 spiked. Each of the extraction methods was used on two controls and two spiked samples and the results were compared.

Samples extracted with the PF method produced an *A*_260/230_ <1.7 and were further purified using the OneStep™ PCR Inhibitor Removal kit (Zymo Research). The control samples extracted using the PS method had a lower concentration than required for sequencing (below 36 ng µl^−1^) and were concentrated using a vacuum concentrator.

A total of 1 µl of the barcoded and pooled library was used for sequencing which contained 89.4 ng of DNA according to Qubit concentration results. Approximately 67 fmol of DNA was used for sequencing.

Sample sequencing was conducted for a total of 45 h until the flow cell showed very few active pores. A total of 13.2 million reads were generated with an average N50 of 720 bp and an estimated 9.48 gigabases (Gb) of sequence produced. A method-wise summary of the sequencing results with the means for each parameter has been listed in Table 2. All additional metadata associated with sequencing has been provided in the supplementary material.

**Table 2.** A summary of concentrations and absorbance ratios between extraction methods These have been noted after the treatment methods (either additional column purification or concentration using a vacuum concentrator). The mean of four samples used for each method has been noted along with the sd within samples.

	PF	PS	**NS**
Concentration (ng µl^−1^)	109.25±11.18	50.15±6.42	51.15±9.23
*A*_260/280_	1.88±0.01	1.83±0.03	1.88±0.04
*A*_260/230_	1.79±0.1	1.95±0.09	2.03±0.18
N50 (bp)	1057±83.3	569.5±73.1	685.5±112.36
Q-score	19.8±0.12	19.2±0.54	19.68±0.1

bp, base pairs; GB, Gigabytes; NS, NucleoSpin® Soil; PF, QIAamp® PowerFecal® Pro DNA kit; PS, DNeasy® PowerLyzer® PowerSoil®.

One of the control sample replicates from the PS method did not sequence well, generating only 3.5 Gb of data. The cause for this difference is unknown as the library for sequencing was prepared with equimolar amounts of each sample.

### Bioinformatic analysis

**Fig. 2.**
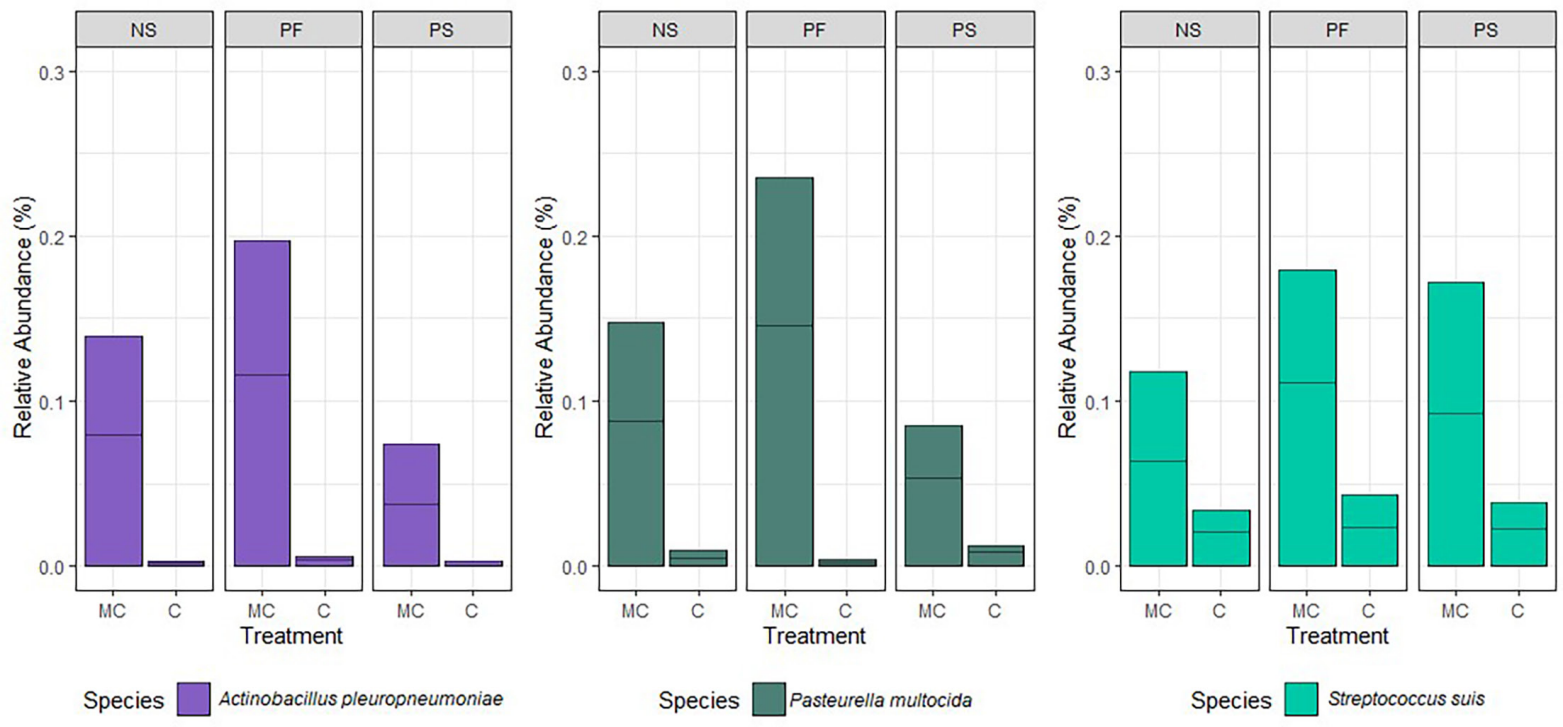
Effect of DNA extraction method on the observed relative abundance of each mock species in spiked wastewater samples. *A. pleuropneumoniae*, *P. multocida* and *S. suis* were spiked into wastewater samples and extracted using three different methods – PF, NS and PS. Two control (un-spiked wastewater) and two spiked samples were extracted by each method. Following extraction, sequencing was conducted using the ONT MinION platform. The relative abundances are based on results from the kraken2 taxonomic classification. The taxonomic classification was analysed using R-studio to produce a relative abundance percentage. MC, Mock Community (Spiked); C, control; PF, QIAamp® PowerFecal® Pro DNA kit; PS, DNeasy® PowerLyzer® PowerSoil®; NS, NucleoSpin® Soil.

Reads under 100 bp were filtered using Filtlong. These filtered reads underwent taxonomic classification using kraken2. The results from kraken2 were analysed using R-studio, and the calculated relative abundances have been represented graphically (Fig. 2). PF showed a consistently higher relative abundance of the three spiked-in bacteria compared to the other two extraction methods.

**Fig. 3.**
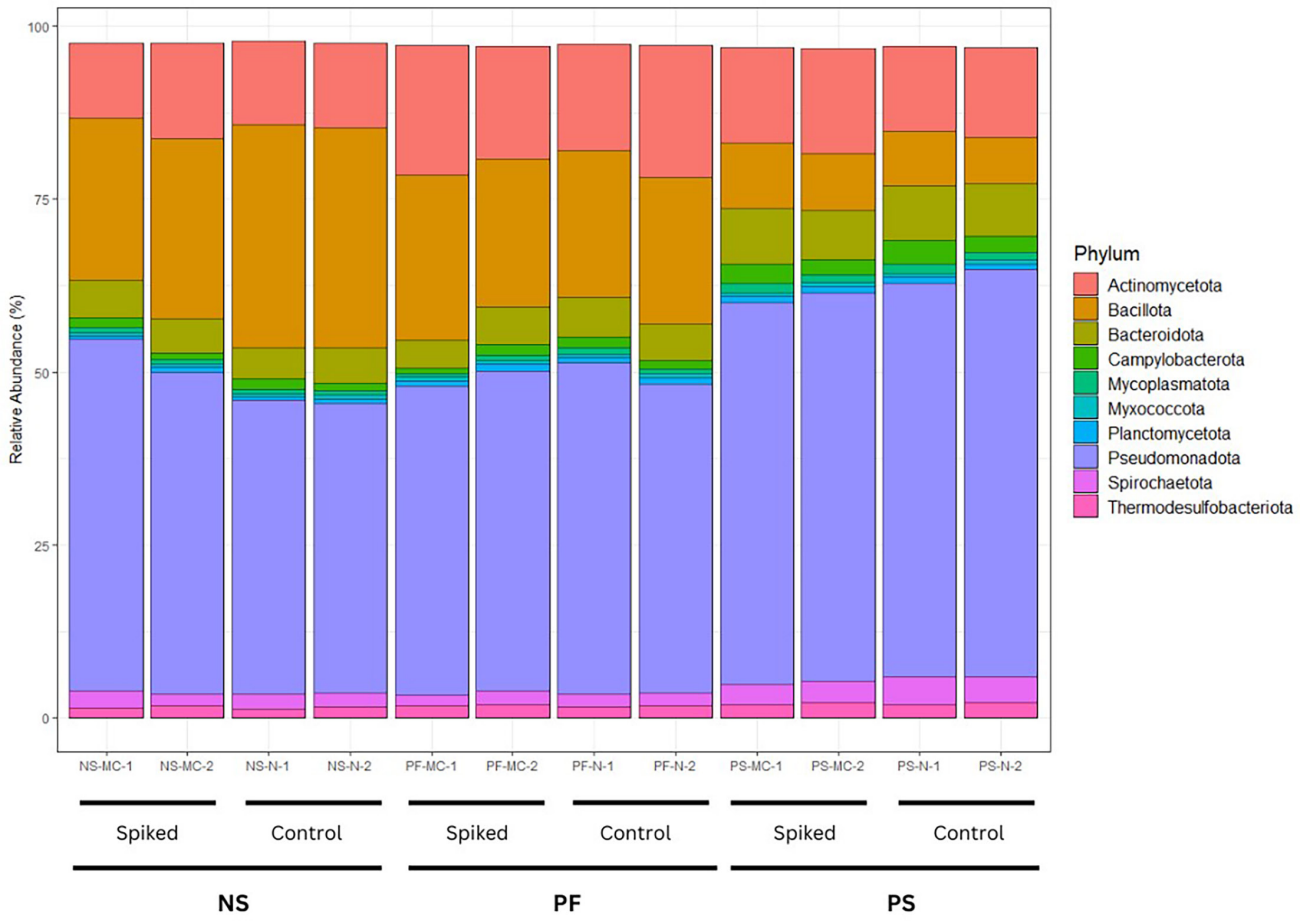
Relative abundance of the top ten phyla recovered from spiked in wastewater samples of farm E, shown by DNA extraction method. Each extraction method was applied to both spiked and unspiked (control) groups. Samples are grouped first by treatment (spiked vs control) and then by extraction method. The relative abundances are based on results from the kraken2 taxonomic classification. The taxonomic classification was analysed using RStudio to produce a relative abundance percentage. PF, QIAamp® PowerFecal® Pro DNA kit; PS, DNeasy® PowerLyzer® PowerSoil®; NS, NucleoSpin® Soil.

**Fig. 4.**
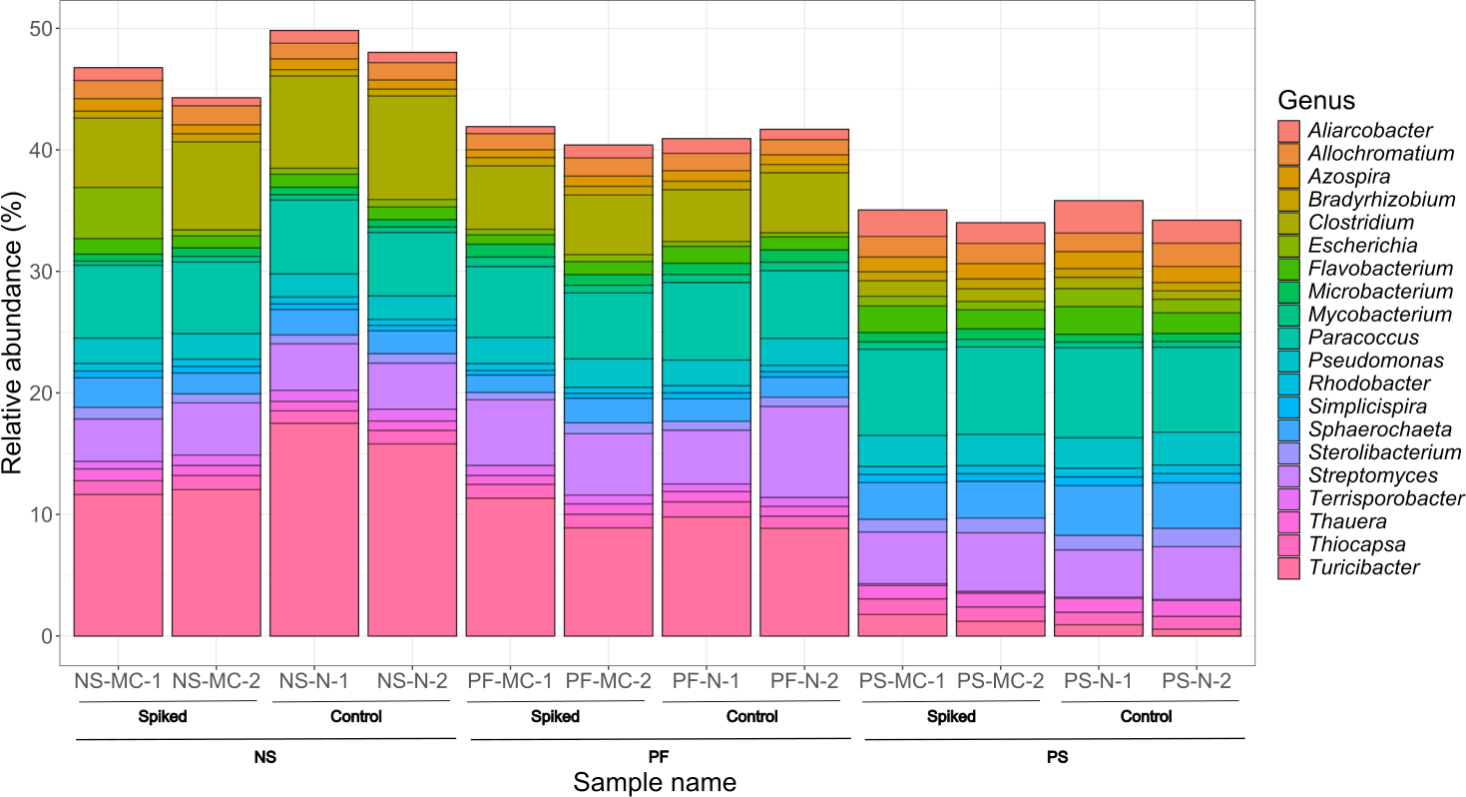
Relative abundance of the top 20 genera recovered from spiked in wastewater samples of farm E, shown by DNA extraction method. Each extraction method was applied to both spiked and unspiked (control) groups. Samples are grouped first by treatment (spiked vs control) and then by extraction method. The relative abundances are based on results from the kraken2 taxonomic classification. The taxonomic classification was analysed using RStudio to produce a relative abundance percentage. PF, QIAamp® PowerFecal® Pro DNA kit; PS, DNeasy® PowerLyzer® PowerSoil®; NS, NucleoSpin® Soil.

The top ten phyla in each of the samples were compared. Samples extracted using the PS method had a higher relative abundance of *Pseudomonadota*, a Gram-negative phylum, but a lower relative abundance of *Bacillota*, a Gram-positive phylum, as compared to samples extracted using the PF and NS methods (Fig. 3). Less significant variations were also observed in *Bacteroidota* (Gram-negative) with PS showing a slightly higher relative abundance. The PF method also showed a higher relative abundance of *Actinomycetota*, a Gram-positive phylum. A comparison was performed at the genus level as well, comparing the top 20 genera in each of the samples (Fig. 4).

**Fig. 5.**
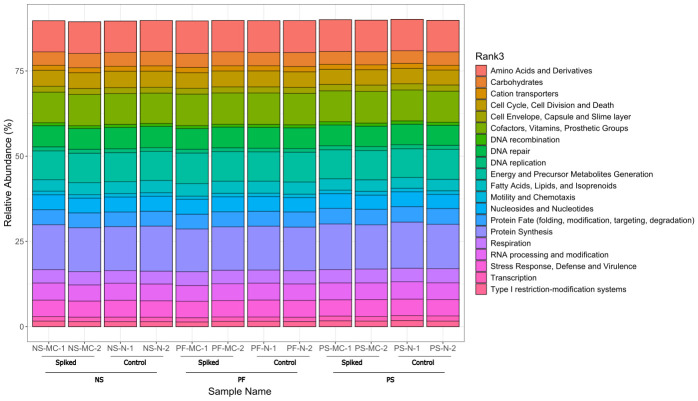
Relative abundance of the top 20 functions recovered from spiked in wastewater samples of farm E, shown by DNA extraction method. Each extraction method was applied to both spiked and unspiked (control) groups. Samples are grouped first by treatment (spiked vs control) and then by extraction method. The functional analysis was performed using DIAMOND+MEGAN7. The SEED was used on MEGAN7 for the functional analysis. PF, QIAamp® PowerFecal® Pro DNA kit; PS, DNeasy® PowerLyzer® PowerSoil®; NS, NucleoSpin® Soil.

In addition to taxonomic comparisons, a functional analysis was performed for each sample to detect any changes in microbiome function observed between extraction methods (Fig. 5).

### qPCR and sequencing comparison

**Fig. 6.**
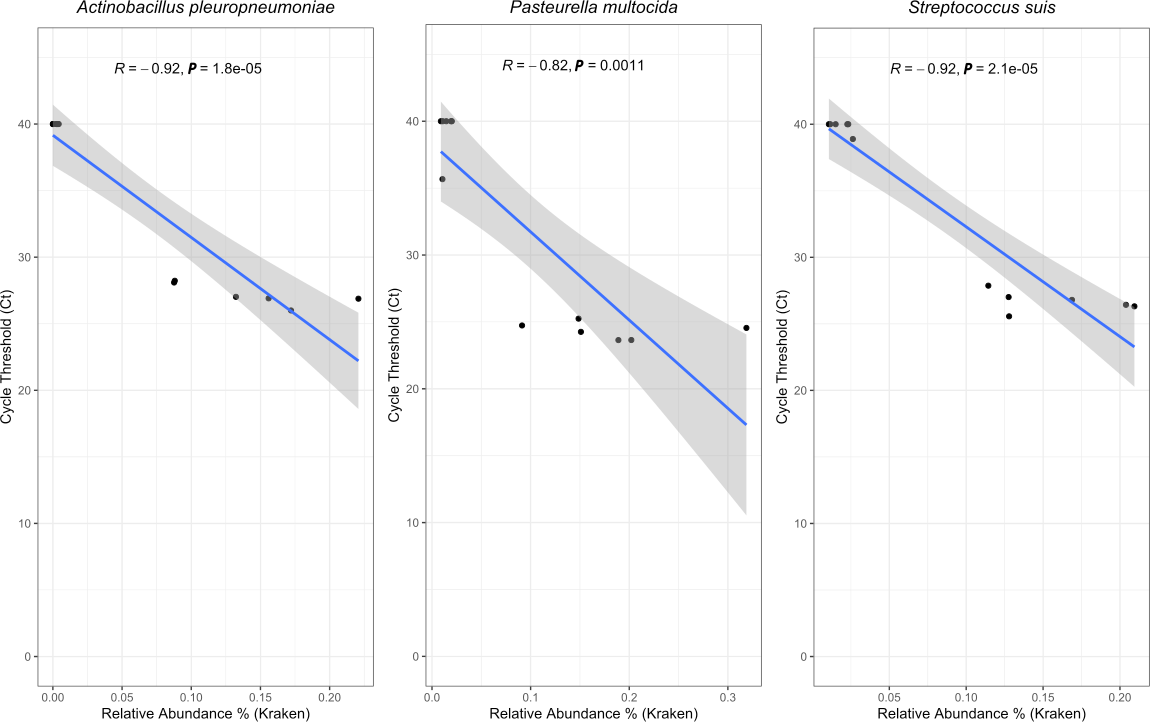
Correlation analysis of Ct values and relative abundance of each species. The graph represents the correlation between relative abundance of each species as determined by kraken2 and the corresponding qPCR Ct values. The correlation was calculated using the non-parametric Spearman method. Each graph notes the Spearman coefficient (R) and the *P*-value. A *P*-value of <0.05 is considered significant.

The microbes of the mock community were successfully detected in all the spiked-in DNA extracts using qPCR. The control samples did not amplify for the microbes of interest, with the exception of a PF control sample which amplified *P. multocida* and a PS control sample which amplified *S. suis*. In both these instances, only one of the two replicates showed amplification, and this amplification had a cycle threshold (Ct) >35 cycles. All three qPCR runs had an *R*^2^ value >0.99 and efficiencies ranged from 86.93 to 96.55, as seen in Fig. 6.

After the qPCR run, the Ct values were correlated with the relative abundances obtained through kraken2 analysis using a non-parametric Spearman correlation. The results of the correlation have been noted in Fig. 4. The Ct values for all the microbes of interest have a strong correlation with the relative abundances. All the correlations were also statistically significant (*P*<0.05).

## Discussion

Effective DNA purification is a vital part of the process for metagenomic sequencing as it influences the composition of the bacterial population obtained through metagenomic analysis [33]. When using metagenomics for pathogen detection, it is very important to consider how the extraction method affects the detection of the bacteria of interest [34]. In this study, we found that extraction method influences the microbial community and plays a role in the detection of low abundance species.

The first requirement of nanopore sequencing is high quality and quantity of extracted DNA. The kits used in this study were evaluated for the quality using absorbance ratios specifically using piggery wastewater. Non-protein contaminants are measured at 230 nm absorbance and a ‘pure’ DNA sample is expected to produce *A*_260/230_ absorbance ratio of 2–2.2. Absorbance at 280 nm measures protein contaminants, and the *A*_260/280_ is expected to fall between 1.8 and 2.0 [35]. Each kit produced variable results in overall yield of extracted DNA. The PM extraction method produced the least amount of DNA and was unsuitable for any downstream analysis due to the presence of particulate matter that could not be settled with centrifugation. The NS kit produced DNA of sufficient quality as indicated by the absorbance values; however, the concentration obtained was consistently lower than that of the PF kit. This result is in contrast with a previous report where the NS kit produced better DNA yields than PF when compared for pig manure and piggery effluent [36], although in both cases the NS kit produced better sample quality than PF. The PS method gave similar results to the NS method, where the quality of DNA obtained was satisfactory, but the quantity was lower than the PF method. The PG kit, while providing enough DNA, failed to produce sufficient quality to be used for downstream ONT sequencing. DNA from the PG and IH methods had extremely low *A*_260/230_ ratios and additionally produced extremely variable yields, suggesting inconsistency among different samples, and was therefore discarded from further experiments. Based on the yield and purity results, we chose the three bead-beating kits (PF, PS and NS) suitable for further studies.

Recovery of known micro-organisms, particularly low-abundance pathogens, can be challenging when spiked in a complex matrix [18]. Therefore, in addition to studying the purity and yield of extraction methods, it is important to evaluate its efficacy on bacteria of interest when present in lower abundance compared to other microorganisms. All three methods reflected a bias towards *P. multocida*, which was spiked in the least quantity but was recovered almost equal to *A. pleuropneumoniae* by all methods. The NS and PF kits also underrepresented *S. suis*, which had a lower relative abundance compared to the two Gram-negative bacteria. Differences in a mock community from expected abundances to actual abundances have been observed previously [37]. The causes for biases can be due to the classification method and database [38], or due to the extraction method itself. Overall, the PF method showed the highest relative abundance of the mock community pathogens, reflecting a good sensitivity for lower abundance bacteria in the matrix.

Based on the results of the relative abundances, the top phyla for each method were compared to examine any potential biases between Gram-positive and Gram-negative bacteria. The most dominant phyla with all extraction methods were *Pseudomonadota*, *Actinomycetota*, *Bacillota* and *Bacteroidota*. In a previous study looking at the communities in pig wastewater at every stage of treatment, the results for dominant phyla are similar [39]. The proportions of dominant phyla varied between extraction methods. Overall, PS had a lower abundance of the Gram-positive phylum *Bacillota* and higher abundance of the two dominant Gram-negative phyla (*Pseudomonadota* and *Bacteroidota*). It is also interesting to note that the PF method had a higher abundance of *Actinomycetota*, a Gram-positive phylum with a rigid cell wall [40], demonstrating an ability to obtain DNA from cells that are difficult to lyse. Since it is difficult to know the reality of the community composition in these samples, it is challenging to conclude which of the methods was closest to reality. However, these results do illustrate the importance of extraction methods in obtaining a good balance between Gram-positive and Gram-negative bacteria in metagenomic studies. When comparing the results at the Genus level, the most distinct differences are observed in the presence of Gram-positive genera *Clostridium* [41] and *Turicibacter* [42] between extraction methods. Both NS and PF have a greater representation of these two genera compared to the PS method. This further illustrates a more thorough lysis of Gram-positive bacteria by PF and NS. There were no differences observed in the functional analysis between methods.

Length of fragments was evaluated using the N50 metric obtained during the sequencing run. The N50 is a read-length value where 50% of the sequenced bases are in reads of this length or longer [43]. The N50 reflected the presence of relatively small DNA fragments ranging from 473 to 1,131 bp across all extraction methods, with the PF method having the longest mean N50. While the read lengths can be compromised using bead beating, it should be considered that given the complexity of wastewater as a matrix, it might be essential to the process. Mechanical lysis in a complex matrix such as a soil sample has been shown to result in more uniform disruption and better penetration of the lysis buffer for downstream metagenomic applications [44]. Bead beating has also been used in activated sludge improving the recovery of Gram-positive bacteria [45].

qPCR was conducted alongside sequencing to provide an additional quantitative measure. In addition to confirming the presence of the mock community in the spiked-in samples, qPCR enabled direct comparisons of quantitative results with the relative abundance estimates from the sequencing. The results obtained from the relative abundance were confirmed by qPCR, where only the spiked samples showed amplification, and the controls did not. The qPCR Ct results were strongly correlated with the relative abundance calculated using kraken2. A study by Ebinger *et al*. [46] showed that the relative abundance of viruses detected using sequencing of different matrices could be correlated with the ng µl^−1^ quantities of the virus as detected by Reverse Transcription-qPCR [46]. However, Andersen *et al*. had diametrically opposite results and concluded that qPCR could not be used for sequencing validation [47]. Based on our study, we conclude that qPCR can be a useful tool to validate metagenomic results for our chosen pathogens.

While we have taken caution in our methodology and replicates, this study does have certain shortcomings. Before conducting sequencing, it would have been useful to conduct fragment analysis to determine the fragment sizes. While we have been able to compare the DNA sizes between methods using the N50, fragment analysis would have been more probative before moving forward with sequencing. In addition, we have made minor modification to the PF method, but not to the other methods. Optimizing a single kit that shows promise can overall enhance its success. It should also be noted that while we explored one non-kit method in our study, there are other options such as phenol-chloroform which has been used with activated sludge [48] and horse faeces [49]. Additionally, the Cetyltrimethylammonium bromide method has been used successfully with gut microbiome samples [50]. These are all methods that should be considered for use with the wastewater in future studies. A follow-up study would also evaluate potential contamination from reagents in the kits which has been noted in literature [51]. In addition to reagent contamination, another form of contamination known as the ‘splashome’ has been noted in literature where samples are contaminated by neighbouring samples during preparation for sequencing [52].

## Conclusions

Based on the results from this study, we identified the PF (QIAamp PowerFecal Pro) method as the most suitable for use with piggery wastewater, specifically with the aim of low abundance pathogen detection.

## Abbreviations

BA/SN: blood agar/selective nutrient
Ct: cycle threshold
DNA: Deoxyribonucleic Acid
ONT: Oxford Nanopore Technology
qPCR: quantitative Polymerase Chain Reaction
SARS-CoV-2: Severe acute respiratory syndrome coronavirus 2
